# Treatment Options for Urogenital Dysfunction in Parkinson’s Disease

**DOI:** 10.1007/s11940-016-0427-0

**Published:** 2016-09-27

**Authors:** Amit Batla, Natalie Tayim, Mahreen Pakzad, Jalesh N. Panicker

**Affiliations:** 1 UCL Institute of Neurology, Queen Square, 7 Queen Square, London, WC1N 3BG UK; 2 Department of Uro-Neurology, National Hospital for Neurology and Neurosurgery, Queen Square, London, UK; 3 Department of Uro-Neurology, The National Hospital for Neurology and Neurosurgery and UCL Institute of Neurology, Queen Square, London, UK

**Keywords:** Lower urinary tract, Detrusor overactivity, Urodynamics, Bladder diary Parkinson’s disease, Erectile dysfunction, Incontinence

## Abstract

Urogenital dysfunction is commonly reported in Parkinson’s disease (PD), and history taking and a bladder diary form the cornerstone of evaluation. The assessment of lower urinary tract (LUT) symptoms include urinalysis, ultrasonography, and urodynamic studies and help to evaluate concomitant urological pathologies such as benign prostate enlargement. Antimuscarinic medications are the first line treatment for overactive bladder (OAB) symptoms and solifenacin has been specifically studied in PD. Antimuscarininc drugs may exacerbate PD-related constipation and xerostomia, and caution is advised when using these medications in individuals where cognitive impairment is suspected. Desmopressin is effective for the management of nocturnal polyuria which has been reported to be common in PD. Intradetrusor injections of botulinum toxin have been shown to be effective for detrusor overactivity, however, are associated with the risk of urinary retention. Neuromodulation is a promising, minimally invasive treatment for PD-related OAB symptoms. Erectile dysfunction is commonly reported and first line treatments include phosphodiesterase-5 inhibitors. A patient-tailored approach is required for the optimal management of urogenital dysfunction in PD.

## Introduction

Parkinson’s disease (PD) is a progressive neurodegenerative disorder characterized by motor complaints of slowness of movements, resting tremor, and gait imbalance; however, “non-motor symptoms” are commonly reported and have a tremendous impact on quality of life [1, 2]. Lower urinary tract (LUT) symptoms are often reported by patients, either *storage symptoms* (urinary urgency, frequency, nocturia, with or without incontinence) or *voiding symptoms* (slow and/or interrupted stream, terminal dribble, hesitancy and straining) [3]. The prevalence of LUT symptoms varies according to the study cited and ranges between 38 and 71 %, being influenced by the stage of disease and presence of LUT-related comorbidities, and parallels other manifestations of autonomic dysfunction [4–7]. The presence of LUT symptoms is associated with an increased risk for falls [8], early institutionalization and escalating health-related costs [9]. Nocturia is the most common non-motor symptom in PD but the causes for nocturia in PD are poorly understood [10, 11].

Urodynamic studies may demonstrate reduced bladder capacity, poor compliance and detrusor overactivity (DO) in 43–93 % of patients of PD patients [12–14]. A likely mechanism for OAB symptoms in PD is disruption of the dopamine D1-GABAergic direct pathway and its GABAergic collateral to the micturition circuit [15–17], resulting in loss of inhibition of the micturition reflex and OAB symptoms. OAB symptoms correlate in severity with urodynamic abnormalities and dopaminergic deficit on dopamine transporter scans [3, 18]. Nocturia additionally may also be due to nocturnal polyuria, characterized by increased nocturnal urine production more than 20–33 % of the entire 24-h volume [19]. Sexual dysfunction (SD) is frequently reported in PD and negatively impacts quality of life of both patients and partners. Men most commonly experience erectile dysfunction (ED) and difficulties in ejaculation, and women report reduced vaginal lubrication, painful intercourse and incontinence during sexual activity [20]. Patients may also report alterations in sexual desire such as hyposexuality or hypersexuality. Motor symptoms of PD such as tremor, muscle rigidity, dyskinesia and bradykinesia can affect sexual performance and satisfaction [20]. The management options for bladder and sexual dysfunction are discussed in this article. This article does not contain any studies with human or animal subjects performed by any of the authors.

### Management of lower urinary tract dysfunction

Despite the high prevalence of LUT symptoms and impact on quality of life, treatment options are currently limited and are often poorly tolerated or ineffective in PD. Most treatment options are derived from guidance around general management of LUT symptoms in neurological patients. Comprehensive history taking is a sound starting point, as this provides insight into whether patients have storage dysfunction or voiding dysfunction, or both. Patients often have other medical comorbidities and the medications prescribed for these may contribute to LUTS, for example, diuretics used for managing hypertension increase urinary urgency and frequency. A review of concomitant medications provides an opportunity to review a patient’s “anticholinergic burden”, and adding an antimuscarinic medication may increase the risk for falls, cognitive impairment and all-cause mortality [21]. Physical examination involves examining the abdomen, flank and pelvic and genital organs, and when appropriate, evaluating urogenital sensations, sacral cord-mediated reflexes (bulbocavernosus reflex, anal reflex) and anal sphincter tone and contractions. Digital rectal examination in a male patient allows evaluation of the size and consistency of the prostate gland.

The bladder diary, ideally maintained by the patient or carer for a period of time usually 3 days, provides prospective information in real-time about LUT symptoms about the fluid intake, urine output, time of voids and recorded indicators of bother and severity of symptoms. It is the only evaluation that provides information about nocturnal polyuria [22••]. Urinalysis (urine dipstick test) provides a rapid method of screening for Urinary tract infections (UTIs) which may share symptoms of OAB in the neurological population [23]. Ultrasonography is a non-invasive test that evaluates the lower and upper urinary tract for pathologies such as stones, hydronephrosis and prostate enlargement and also provides a measure of the post-void residual urine [24••, 25, 26••]. Uroflowmetry is a non-invasive urodynamic study that provides information about voiding. It can be used to diagnose bladder outflow obstruction and assess the patient’s flow rate, time and pattern. Cystometry is not performed as a routine, however, may be required to evaluate the cause for LUT symptoms and exclude concomitant pathologies such as bladder outlet obstruction (Table 1) [24••, 25, 26••].

**Table 1:** The clinical assessment of urogenital dysfunction in PD (modified from Panicker et al. 2015 [27••])

	Bedside evaluation	Non-invasive tests	Invasive tests
Essential	History taking; physical examination; bladder diary	Urinalysis; post-void residual urine volume measurement; ultrasonography	–
Desirable	Questionnaires	Uroflowmetry; blood biochemistry	–
Required in specific situations	–	Urine culture; urine cytology Hormone profile (testosterone), penile Doppler	(Video-)urodynamics; flexible cystoscopy; pelvic neurophysiology; renal scintigraphy

## Diet and lifestyle

Although there are no studies specifically in PD, lifestyle interventions and behaviour modifications can improve OAB symptoms in patients with neurological disorders. Caffeine and/or alcohol should be avoided where possible as these both have mild diuretic properties. Restricting fluid intake at least 4 h prior to bedtime may help with nocturia [28]. Absorption of dependent edema fluid at night may contribute to nocturia, and adequate exercise, lower limb elevation above the heart level in the afternoon and use of compression stockings may help address this. The use of diuretics in the late afternoon may help with nocturia [29]. Emptying the bladder before going to bed is also advisable. To reduce the risk for falls at night, the path to the toilet should be well-lit and the area should be made ‘falls safe’ to avoid falls and injuries. In patients with limited mobility but preserved continence, simple measures such as using a bedside commode or flask may obviate the need to walk to the toilet at night [30•].

## Pharmacologic treatment

### Antimuscarinic agents

- Antimuscarinic agents are the first line treatment for OAB symptoms [31]. These include oxybutynin, tolterodine, solifenacin, darifenacin, fesoterodine and trospium chloride [27••, 32] (Table 2).

**Table 2:** Drugs used for management of LUT symptoms in PD

Name	Dose in mg	Frequency	Evidence for use in neurogenic LUT dysfunction	Evidence for use in PD
Antimuscarinic drugs				
Darifenacin-controlled release	7 · 5–15	Once daily	NA	NA
Fesoterodine-controlled release	4–8	Once daily	NA	NA
Oxybutynin			Level 1	Level 5
Immediate release	2 · 5–5	Two or three times a day		
Controlled release	5–20	Once daily		
Transdermal patch	36 (releasing ∼3 · 9 mg oxybutynin per 24 h)	Replace once every 3–4 days		
Solifenacin-controlled release	5–10	Once daily	Level 2	Level 2
Tolterodine			Level 3	Level 5
Immediate release	2–4	Once or twice daily		
Controlled release	4	Once daily		
Trospium chloride			Level 1	Level 5
Immediate release	20	Twice daily (before food)		
Controlled release	60	Once daily		
Other drugs				
Mirabegron	25 to 50 mg	Once daily	NA	NA
Desmopressin			NA	Level 5
Nasal spray	5 to 40 mcg/day	Once daily		
Tablets	0.1 mg	Once daily		
Injections	4 mcg/mL	Once daily
- Antimuscarinic agents competitively antagonize muscarinic acetylcholine receptors, prevent detrusor contraction, lower intravesical storage pressures and reduce storage symptoms.
  The effect that these medications may have on central muscarinic receptors has attracted considerable interest in recent years [24••, 33]. The central effects of these medications may result in alterations in cognition and consciousness in susceptible individuals. Medications currently being used by the patient should be reviewed before prescribing an antimuscarinic agent, especially in older people, as the cumulative use of agents with anticholinergic properties is associated with increased risk of cognitive impairment [33]. Permeability across the blood–brain barrier differs between antimuscarinic agents based upon physicochemical properties. For example, trospium chloride is a poorly lipophilic, positively charged quaternary ammonium compound that does not readily cross the blood–brain barrier and theoretically might be associated with fewer effects on cognition, though this has not been specifically evaluated in PD [34].
- Muscarinic receptor selectivity may influence central effects. For example, darifenacin has a greater affinity for the M3 receptor subtype of relevance to bladder functions compared to the M1 receptors prevalent in the central nervous system A recent double-blind, randomized, placebo-controlled study evaluated solifenacin in 23 patients with PD and urinary symptoms. There was no significant change in the mean number of micturition per 24 h period during the double-blind phase, but in the open label extension phase there was an improvement in the number of micturitions and number of nocturia episodes per 24 h period at a mean dose of 6 mg/day [35••].

#### Darifenacin [36]

**Standard dosage**: 7. 5 to 15 mg once daily.
**Class of evidence**: NA [24••, 37]
**Contraindications**: Urinary retention; narrow angle glaucoma; gastric or intestinal obstruction, severe dementia or myasthenia gravis.
**Main drug interactions**: Preparations containing potassium can increase gastric intolerance, topiramate or zonisamide can increase chances of heat prostration.
**Main side effects**: Constipation, headache and xerostomia. Other side effects include urinary retention or tract infection and blurred vision. Anticholinergic central nervous system (CNS) effects are specifically relevant in elderly and patients with PD dementia.
**Special points**: Safety during pregnancy uncertain [Category C].

#### Fesoterodine [36]

**Standard dosage**: 4–8 mg once daily.
**Class of evidence**: 5 [24••, 37]
**Contraindications**: Urinary retention; narrow angle glaucoma; gastric or intestinal obstruction, severe dementia or myasthenia gravis [38].
**Main drug interactions**: Preparations containing potassium can increase gastric intolerance, topiramate or zonisamide can increase chances of heat prostration.
**Main side effects**: Xerostomia (19–35 %), headache insomnia and constipation. Other side effects include urinary retention or tract infection and blurred vision. Anticholinergic CNS effects are specifically relevant in elderly and patients with PD dementia.
**Special points**: Safety during pregnancy uncertain [Category C].

#### Oxybutinin [36]

**Standard dosage**: Immediate release tablet: 2.55 mg two to three times daily.
**Extended release tablets**: 5–20 mg daily.
**Transdermal patch**: 3.9 mg/day, applied twice weekly (every 3 to 4 days).
**Class of evidence**: Level 5 for urinary dysfunction in PD [11, 37] [level 1 evidence for neurogenic detrusor overactivity [39]]
**Contraindications**: Urinary retention; narrow angle glaucoma; gastric or intestinal obstruction, severe dementia or myasthenia gravis [38].
**Main drug interactions**: Preparations containing potassium can increase gastric intolerance, topiramate or zonisamide can increase chances of heat prostration.
**Main side effects**: Constipation, headache and xerostomia. Other side effects include urinary retention or tract infection and blurred vision. Anticholinergic CNS effects are specifically relevant in elderly and patients with PD dementia.
**Special points**: Pregnancy category B.

#### Solifenacin [36, 40]

**Standard dosage**: 5–10 mg orally per day
**Class of evidence**: Level 1a for urinary dysfunction in PD [35••] (level 1 evidence for neurogenic detrusor overactivity [40].
**Contraindications**: Urinary retention; narrow angle glaucoma; liver disease; kidney disease; intestinal obstruction; long QT syndrome.
**Main drug interactions**: Ketoconazole may significantly increase the blood levels of solifenacin, clozapine, citalopram, clarithromycin and other drugs that can prolong QT interval can increase the risk of arrhythmia with solifenacin
**Main side effects**: Constipation and xerostomia. Other side effects include blurred vision and heat intolerance.
**Special points**: Safety during pregnancy uncertain [Category C], reduced dose (5 mg/day) possible in hepatic and renal failure

#### Tolterodine [36, 38]

**Standard dosage**: 2 mg twice a day or 4 mg extended release capsule
**Class of evidence**: Level 5 for urinary dysfunction in PD [37, 41•] [level 3 evidence for neurogenic detrusor overactivity [42]]
**Contraindications**: Urinary retention; narrow angle glaucoma; gastric or intestinal obstruction, severe dementia or myasthenia gravis [38].
**Main drug interactions**: Preparations containing potassium can increase gastric intolerance, topiramate or zonisamide can increase chances of heat prostration.
**Main side effects**: Constipation, dyspepsia, dizziness and xerostomia. Other side effects include urinary retention or tract infection and blurred vision. Anticholinergic CNS effects are specifically relevant in elderly and patients with PD dementia.
**Special points**: Safety during pregnancy uncertain [Category C].

#### Trospium chloride [36, 43]

**Standard dosage**: 20 mg twice a day or 60 mg extended release capsule
**Class of evidence**: Level 5 for urinary dysfunction in PD [24••, 37] [level 1 evidence for neurogenic detrusor overactivity [43]]
**Contraindications**: Urinary retention; narrow angle glaucoma; gastric or intestinal obstruction, severe dementia or myasthenia gravis.
**Main drug interactions**: Preparations containing potassium can increase gastric intolerance, topiramate or zonisamide can increase chances of heat prostration.
**Main side effects**: Constipation, headache and xerostomia. Other side effects include urinary retention or tract infection and blurred vision. Anticholinergic CNS effects are specifically relevant in elderly and patients with PD dementia.
**Special points**: Safety during pregnancy uncertain [Category C].

#### β3-Adrenoceptor agonists

##### (Mirabegron) [44, 45]

- Recently, mirabegron has emerged as a potentially safe and effective treatment for OAB [44] with preliminary evidence in neurogenic OAB, though not specifically in PD [45]
- However, high-level evidence supporting the use of mirabegron in the management of the neurogenic bladder is lacking.

**Standard dosage**: 25 to 50 mg per day
**Class of evidence**: NA [44]
**Contraindications**: Patients with severe uncontrolled hypertension (BP > 180/110 mmHg) [46]
**Main drug interactions**: Tamoxifen levels may be reduced in patients taking this for breast cancer
**Main side effects**: Worsening of palpitation, preexisting hypertension and urinary retention
**Special points**: Safety during pregnancy uncertain [Category C].

#### Desmopressin

- Desmopressin, a synthetic analogue of arginine vasopressin, temporarily reduces urine production and volume-related OAB symptoms. Desmopressin works by promoting water reabsorption at the distal and collecting tubules of the kidney. It is useful for the treatment of urinary frequency or nocturia.
- Desmopressin has been tried in parkinsonism including PD and multiple system atrophy; however, long-term follow-up is lacking [38, 48, 47•, ].
  **Standard dosage**: Nasal spray 5 to 40 mcg/day tablets 0.1 mg, injections 4 mcg/mL
  **Class of evidence**: Level 5 [48]
  **Contraindications**: Kidney disease, cystic fibrosis, hyponatremia, heart failure and pedal edema. Used with caution over 65 years of age.
  **Main drug interactions**: Antidepressants—citalopram, sertraline and duloxetine can increase chances of hyponatremia with desmopressin
  **Main side effects**: Headache, hyponatremia specifically relevant in patients older than 65.
  **Special points**: Safety during pregnancy uncertain [Category C].

## Botulinum toxin

Intradetrusor injection of onabotulinumtoxinA has proven to be a safe and effective treatment for neurogenic DO. In one study of 20 patients with DO moderate to marked symptom relief at 3 months and a 50 % incontinence decrease over 6 months relative to pretreatment was reported in 59 % patients (*p* ≤ 0.02) [49••]. Another study of eight patients reported clinical and urodynamic improvement in overactive bladder symptoms that lasted at least 6 months in patients with PD [50]. In another study, Kulacksizoglu et al. reported no increase in post-voiding residual [51]. OnabotulinumtoxinA treatment results in impaired detrusor contractility and therefore is associated with a risk for urinary retention. The potential benefits of this treatment must be weighed against the potential need to intermittently catheterize or use an indwelling catheter, and the accompanying dependence on carers or partners and possible complications including urinary tract infection, trauma, bleeding, potential for lower limb weakness and flu-like illness.

## Assistive procedures

### Clean intermittent self catheterization

The finding of a high post-void residue (PVR) is unusual in patients with PD. If the PVR volume is consistently more than 100 mL, clean intermittent catheterization has been advocated. Specific issues related to dexterity in PD may make this challenging. Experienced health-care professionals, such as a continence adviser, should be involved in teaching the technique and exploring possible barriers to successful catheterization. Complications include UTI and trauma [26••, 52•].

## Neuromodulation

- Percutaneous tibial nerve stimulation (PTNS) is an emerging treatment option that may help patients who have contraindications or are refractory to medical treatment. Following acute PTNS, the functional bladder capacity has been shown to be increased [53]. Chronic stimulation has been reported to decrease frequency, urinary and urge urinary incontinence in neurogenic bladder, but long term outcomes in PD are lacking [53]
- Transcutaneous tibial nerve stimulation (TTNS) was found effective in the treatment of LUT symptoms in 13 patients with PD, reducing urgency and nocturia [54]
- Sacral neuromodulation (SNM) may be effective and safe for the treatment of patients with neurogenic lower urinary tract dysfunction (LUTD). However, studies specifically in PD are lacking. A systematic review and meta-analysis included only four patients with PD and this group was not separately analysed. [55].

## Specific therapies for Parkinson’s disease i﻿n management of urological dysfunction

### Dopaminergic treatment

The effects of levodopa on LUT symptoms are variable [3, 56, 57]. There may be a period of initial worsening after starting levodopa but this may improve later [3]. One study showed that treatment with levodopa for 3 months in PD patients was associated with slightly improved storage urodynamic parameters [3]. In another study, DO was shown to worsen with levodopa in some patients and lessened in others. The effect on micturition of treatment with dopaminergic drugs in PD was unpredictable in another study [56]. Dopaminergic drugs like bromocriptine can lead to decreased storage parameters and pergolide improved nocturia in a small study [57].

In an open label study including three patients 12 weeks of treatment with pergolide improved LUT symptoms and nocturia frequency in all three patients and an improvement of sleep QOL in two [57].

### Deep brain stimulation

DBS (DBS) is a well-accepted treatment option in advanced PD. Deep brain stimulation can have variable effects on LUT symptoms with worsening reported in some and improvement in other studies [58, 60, 59••, ]. Although the studies have not specifically focused on nocturia, there is evidence that subthalamic nucleus DBS-treated patients exhibit significantly less nocturia. In a study comparing DBS (GPi) and other forms of treatment in 107 patients with PD, the overall amount of urinary symptoms were similar but fewer DBS patients (47 %) complained of nocturia compared to conventionally treated (88 %) and to apomorphine pump-treated (66 %) patients [61]. Not only did the patients who had DBS reported less nocturia (*P* = 0.007) but also they were also less bothered by nocturia (*P* = 0.01) compared to the other treatment groups, as assessed by the Danish Prostate Symptom Score (DanPSS) [61]. More studies may be needed to establish definite correlation and to understand the pathophysiology of this association.

## Surgical therapies for bladder outflow obstruction

Benign prostate enlargement is a common cause for bladder outflow obstruction in middle age and elderly men and is often a contributory factor for LUT dysfunction in PD. It was widely believed for several years that men with PD should not undergo prostate surgery because of the high risk of incontinence [62]. There is some evidence to support transurethral prostate resection for bladder outflow obstruction in patients with PD. In a study on 23 patients with PD, TURP was successful in up to 70 %. The risk of de novo urinary incontinence after surgery was reported as minimal [63••].

## Management of sexual dysfunction

Management of sexual dysfunction (SD) in patients with PD includes both behavioural and pharmacological options depending on the nature of the sexual dysfunction. Behavioural therapy may be used to treat SD, if considered as a learned maladaptive behaviour [64] and may involve the use of psychodynamic psychotherapy and cognitive behavioural therapy [64, 65••]. Pharmacological treatment of SD, on the other hand, requires either the reduction or elimination of drugs interfering with the sexual function or the introduction of drugs that improves sexual function [64, 65••]. Ultimately, treatment options for SD may require multidisciplinary input from neurologists and psychologists for optimum results [64]. Although phosphodiesterase 5 inhibitors (below) are standard treatment option for erectile dysfunction [66], intracavernosal alprostadil (prostaglandin E2) 1.25–10 μg injections can be used.

The management of hypersexuality as part of an impulse control disorder includes reduction/stopping of dopamine receptor agonist and practical therapeutic strategies including psychological therapies but not limited to counselling, psychotherapy, sex, couple and behavioural therapies [65••]. Hormonal treatment specifically testosterone has been tried in PD [67].

### Sildanefil

**Standard dosage**: 25–100 mg taken 1 h before sexual activity
**Class of evidence**: Level 5
**Contraindications**: Hypotension (blood pressure below 90/50 mmHg)
**Main drug interactions**: Organic nitrates in any form, alpha-blockers and antihypertensives may cause hypotension.
**Main side effects**: Headache, flushing and dyspepsia. Temporary visual symptoms (mainly colour-vision disturbances) may occur with higher doses 100 mg
**Special points**: Used once or twice per week.

### Tadalafil

**Standard dosage**: 10 mg orally once a day, as needed, prior to sexual activity
**Class of evidence**: Level 5
**Contraindications**: Hypotension (blood pressure below 90/50 mmHg)
**Main drug interactions**: Organic nitrates in any form, alpha-blockers and antihypertensives may cause hypotension.
**Main side effects**: Headache, flushing and dyspepsia. Temporary visual symptoms (mainly colour-vision disturbances) may occur with higher doses 100 mg
**Special points**: Used once or twice per week.
